# Supplementary material for: Feasibility of EBUS-TBNA for histopathological and molecular diagnostics of NSCLC—A retrospective single-center experience
Source: PLoS One. 2022 Feb 2;17(2):e0263342. doi: 10.1371/journal.pone.0263342 (PMC8809531; doi:10.1371/journal.pone.0263342)
Supplement: S1 File — Note that for NGS and PCR the best material based on tumor cell content and fraction was selected regardless of specimen type. (DOCX) [file pone.0263342.s001.docx]

**Supplementary Table 1**. Routine mutation, fusion, and PD-L1 analysis at different times (the years of inclusion in the study was 01/01/2017-23/04/2018)

| Time period | Mutation analysis – first choice | Mutation analysis – backup/limited material | Fusion analysis – first choice | Fusion analysis – backup/limited material | PD-L1 |
| --- | --- | --- | --- | --- | --- |
| Until 03/2018 | NGS Ion AmpliSeq™ Colon and Lung Panel v2 | Therascreen® *EGFR* RGQ PCR | Histology (preferred): IHC for ALK and ROS1 (FISH for confirmation)  Cytology: FISH for ALK and ROS1 | - | Histology preferred, IHC clone 28-8 (Abcam) until 12/2017, 22C3 (Agilent/pharmDx) from 01/2018 |
| From 03/2018 | NGS Oncomine™ Focus Assay | Therascreen® *EGFR* RGQ PCR | NGS Oncomine™ Focus Assay | Histology (preferred): IHC for ALK and ROS1 (FISH for confirmation)  Cytology: FISH for ALK and ROS1 | Histology preferred, IHC 22C3 (Agilent/pharmDx) |

Note that for NGS and PCR the best material based on tumor cell content and fraction was selected regardless of specimen type
